# Target Amplification and Distractor Inhibition: Theta Oscillatory Dynamics of Selective Attention in a Flanker Task

**DOI:** 10.3758/s13415-021-00876-y

**Published:** 2021-03-15

**Authors:** Céline C. Haciahmet, Christian Frings, Bernhard Pastötter

**Affiliations:** University of Trier, Universitätsring 15, 54286 Trier, Germany

**Keywords:** Attention, Cognitive control, Midfrontal theta, Target amplification

## Abstract

Selective attention is a key mechanism to monitor conflict-related processing and behaviour, by amplifying task-relevant processing and inhibiting task-irrelevant information. Conflict monitoring and resolution is typically associated with brain oscillatory power increase in the theta frequency range (3-8 Hz), as indexed by increased midfrontal theta power. We expand previous findings of theta power increase related to conflict processing and distractor inhibition by considering attentional target amplification to be represented in theta frequency as well. The present study (*N* = 41) examined EEG oscillatory activities associated with stimulus and response conflict in a lateralized flanker task. Depending on the perceptual (in)congruency and response (in)compatibility of distractor-target associations, resulting stimulus and response conflicts were examined in behavioural and electrophysiological data analyses. Both response and stimulus conflict emerged in RT analysis. Regarding EEG data, response-locked cluster analysis showed an increase of midfrontal theta power related to response conflict. In addition, stimulus-locked cluster analysis revealed early clusters with increased parietal theta power for nonconflicting compared to conflicting trials, followed by increased midfrontal theta power for both stimulus and response conflict. Our results suggest that conflict resolution in the flanker task relies on a combination of target amplification, depicted by parietal theta power increase, and distractor inhibition, indexed by midfrontal theta power increase, for both stimulus and response conflicts. Attentional amplification of sensory target features is discussed with regard to a domain-general conflict monitoring account.

## Introduction

Selective attention helps to focus on relevant information in the environment, while ignoring distracting events, thus serving as an important device for cognitive control and enabling humans to pursuit goal-directed behaviour. Changes in environmental demands need to be detected rapidly by our conflict monitoring system, so we can allocate more attention and cognitive resources to a prioritized task, while simultaneously suppressing distracting information (Botvinick, Cohen, & Carter, 2004). It is still an open debate whether top-down conflict resolution relies more on attentional target amplification, inhibition of irrelevant information, or a combination of both strategies (Frings, Schneider, & Fox, 2015; Nigbur, Schneider, Sommer, Dimigen, & Stürmer, 2015).

Up to now research has focused on neural processing of conflict management and top-down attentional control in the anterior cingulate cortex (ACC; Botvinick, Braver, Barch, Carter, & Cohen, 2001) and prefrontal cortex (Corbetta & Shulman, 2002). In the electroencephalogram EEG, conflict processing is typically associated with a power (or amplitude) increase in the theta frequency range, as indexed by increased midfrontal theta power (around 6 Hz) originating from the ACC (Nigbur, Cohen, Ridderinkhof, & Stürmer, 2012), accompanied by slower reaction times and more errors in conflicting conditions. In addition, attentional processing of distractors has been linked to increased theta power over parietal sites (Pastötter & Frings, 2018). Going beyond these theta power effects, in the present study, we examined whether attentional amplification of target stimuli also is linked to theta power increase, accompanied by faster reaction times and less errors in non-conflicting conditions. To test this hypothesis, we examined behavioural and EEG theta power effects in a modified version of the flanker task (Eriksen & Eriksen, 1974) to induce cognitive conflict, namely stimulus conflict and response conflict.

### Cognitive conflict in the Flanker Task

Conflict arises when two or more stimulus dimensions are inconsistent and when one of the processes interferes with the internal goal to follow task instructions (Oehrn et al., 2014). Stimulus conflict emerges at the perceptual level when target information differs in perceptual qualities from distractor information. The resulting stimulus-stimulus incongruency affects processing at an early sensory processing level (Merz, Frings, & Spence, 2020). In contrast, response conflict operates at the level of response selection, when the human cognitive system needs do differentiate between compatible and incompatible response options (Cohen & Cavanagh, 2011).

A well-established paradigm to investigate the processing of stimulus and response conflicts is the Flanker Task. Eriksen and Eriksen (1974; for a recent review see Merz et al., 2020) developed a paradigm to originally study visual information processing of a central target letter flanked by a number of noise letters (distractors). Participants are instructed to make a speeded discriminative response to the target’s identity with one of two different spatial responses in most cases (e.g., left or right; Gratton, Coles, & Donchin, 1992). The emerging conflict in the flanker paradigm evokes two additive subcomponents: stimulus and response conflict. On the one hand, incongruent stimulus features between target and distractors lead to stimulus conflict at the perceptual level (referring to incongruent S-S overlap according to Kornblum´s taxonomy; Kornblum, Hasbroucq, & Osman, 1990). On the other hand, target and distractors may be mapped to different response keys, which creates response conflict at the response selection level. The flanker-compatibility effect refers to the interference caused by response incompatible distractors, whereas the flanker-congruency effect refers to the interference caused by conflicting sensory features between target and distractor stimuli (Hommel, 2004).

### Neural mechanisms of cognitive control

The neural correlates of conflict management and executive control in general often are ascribed to a network consisting of regions in the prefrontal cortex (PFC) and especially the ACC (Fan, Flombaum, McCandliss, Thomas, & Posner, 2003; Yeung, Botvinick, & Cohen, 2004). For instance, the conflict monitoring account (Botvinick et al., 2001) states that the ACC monitors and detects possible conflicts and signals the need for top-down control implementation to prefrontal cortex areas, such as dorsolateral PFC. Botvinick et al. (2001) suggested that the ACC serves an evaluative function by detecting conflict in a first step, followed by biasing the processing of task-specific dimensions and suppressing task-irrelevant dimensions in further steps. Consistently, with regard to EEG (and magnetoencephalography, MEG, and intracranial EEG), power changes in prefrontal theta activity (4-8 Hz) have been linked to temporal organization of neural processes around decision points, such as action selection and action monitoring (Cavanagh, Zambrano-Vazquez, & Allen, 2012). Increases in midfrontal theta power and theta phase synchronization between PFC regions and ACC have been associated with conflict detection, response monitoring, and cognitive control (Oehrn et al., 2014). Typically, oscillatory analyses of EEG and MEG data in conflict tasks observed prominent effects of both stimulus and response conflicts in midfrontal theta power (Cohen & Donner, 2013; Duprez, Gulbinaite, & Cohen, 2018; Nigbur et al., 2012; Pastötter, Dreisbach, & Bäuml, 2013), likely originating from the ACC (Cavanagh et al., 2012; Cavanagh & Shackman, 2015; Onoda, Kawagoe, Zheng, & Yamaguchi, 2017). In addition, van Driel, Swart, Egner, Ridderinkhof, & Cohen (2015) reported interregional theta phase synchrony between midfrontal, lateral frontal and posterior parietal regions in order to direct top-down conflict control (see also Hanslmayr et al., 2008; Oehrn et al., 2014). The functional connectivity in theta band between medial, frontal, and parietal regions may reflect the long-range communication in the cognitive conflict network that was postulated by Botvinick et al. (2001).

In addition to PFC and ACC, it is a prominent view that, in non-conflict tasks (e.g., attention-cuing tasks), the parietal cortex mediates (or even initiates) attentional control and the processing of attended stimulus information (Corbetta & Shulman, 2002; Friston & Büchel, 2000; Kastner & Ungerleider, 2000). With regard to EEG theta oscillations, Green & McDonald (2008), for instance, examined attentional shifts to task-relevant stimuli in a spatial cuing paradigm and found that early parietal cortex activation initiates the signal for attentional control and conveys it to frontal cortices. Specifically, the authors reported that they observed theta activity in the parietal cortex 100–200 ms before theta activity in the frontal cortex. In addition, the magnitude of early parietal activation was strongly predictive of the degree of attentional improvement in perceptual performance, i.e., perceptual accuracy of target discrimination. These findings suggest that early parietal theta power increase is related to enhanced processing of nonconflicting visual target information. Empirical evidence for such association between (early) parietal theta power increase and attentional amplification of (nonconflicting) target information, however, is less clear for experimental paradigms in which cognitive conflict, i.e., stimulus or response conflict, of distractor information arises, as it does in the Eriksen flanker task.

### Attentional distraction to conflicting information

Pastötter and Frings (2018) recently demonstrated that early sensory conflict processing of distractors can precede conflict-related midfrontal theta power increase in a lateralized single-flanker task. The authors investigated a sensory lateralization effect in EEG theta power, which refers to hemifield-specific perceptual processing of distractor information in the parieto-occipital cortex. The key concept is that attention modulates perception in a spatially selective way, such as that lateral presented distractors receive enhanced attentional processing in the contralateral hemisphere. Pastötter and Frings (2018) found a prominent sensory lateralization effect in theta power that was modulated by response conflict. Both evoked and induced theta power over occipital electrodes showed a stronger lateralization effect in incompatible compared to compatible trials. The authors argued that due to common neural coding of stimulus and response features in event files, early modulation of sensory distractor processing, indexed by sensory theta lateralization, indeed can be induced by response conflict. This view is consistent with the Theory of Event Coding (TEC; Hommel, 2019; Hommel, Müsseler, Aschersleben, & Prinz, 2001) and the Binding-and-Retrieval-in-Action-Control (BRAC) framework (Frings et al., 2020). Because the sensory lateralization effect was negatively related to the conflict-related midfrontal theta power effect, Pastötter and Frings (2018) argued that the parietal cortex initiates attentional control and communicates conflicting information to frontal sites (see also Vissers, Ridderinkhof, Cohen, & Slagter, 2018, for related findings).

Consistently, an event-related potential (ERP) study by Appelbaum et al. (2011) provided evidence for conflict-induced interaction between frontal and parietal sites in the flanker task. The authors demonstrated a conflict-related sensory lateralization effect in the N200 component, which might be attributed to enhanced lateralisation of evoked theta power due to (a combination of stimulus and response) conflict. The authors concluded that stimulus incongruent distractors cause distraction and attention allocation in parieto-occipital areas. Thus, there is recent evidence for an association between (both evoked and induced) parietal theta power and attentional distraction of interfering information. Just like the prominent midfrontal conflict effect, the sensory lateralization effect is reflected by a relative increase of theta power in conflict trials in comparison to non-conflict trials. Such increase of theta power in conflict trials is referred to as a *positive theta effect* in the following. In contrast, if there is an association between parietal theta power increase and attentional amplification of target information in the flanker task, such effect should be reflected by a relative decrease of theta power in conflict compared to non-conflict trials, which is referred to as *negative theta effect* in the following.

### Brain-behaviour correlations in theta frequency

In the literature, reported findings are ambiguous with regard to the correlation between conflict-related midfrontal theta power and behavioural conflict-related effects, i.e., increase of reaction time and decrease of accuracy. Although theta oscillations seem to have an active and causal role in shaping behaviour (Lehr, Henneberg, Nigam, Paulus, & Antal, 2019; van Driel, Sligte, Linders, Elport, & Cohen, 2015), the empirical landscape reports inconsistent correlations between conflict effects on midfrontal theta power and reaction time. Indeed, positive correlations (Cohen & Donner, 2013; Green & McDonald, 2008; Pastötter et al., 2013), negative correlations (Oehrn et al., 2015; Pastötter, Hanslmayr, & Bäuml, 2010), and zero-order correlations (Pastötter & Frings, 2018; Zavala et al., 2013) have been observed. This inconsistent pattern of results suggests that variance in conflict-induced theta power reflects yet undefined processes that go beyond conflict management in the PFC (see Carp et al., 2010; van Driel et al., 2015). Indeed, positive theta effects related to the processing of distractor information and negative theta effects related to the processing of target information, both being entangled in the cognitive conflict network, might shape behaviour in interdependent ways. Thus, behavioural effects in conflict tasks might be differentially related to distractor inhibition (Zavala et al., 2013), reflected in positive theta effects, and attentional target amplification reflected in negative theta effects.

### The present study

The main goal of the present EEG experiment was to go beyond the study of conflict management in midfrontal theta power and examine attentional amplification processes related to theta power in a lateralized version of the flanker task. Thus, we maintained a domain-general conflict resolution account that combines various strategies, including distractor suppression and enhanced target processing, both relying on their common neural coding in the theta (3-8 Hz) frequency range. Both stimulus and response conflict effects were investigated. Conflict effects in EEG theta power were examined both time-locked to the onset of stimuli and time-locked to the onset of responses. We had the following hypotheses: First, we expected to replicate prominent midfrontal theta power increase (i.e., a *positive theta effect*) for both stimulus and response conflicts (Cavanagh et al., 2012; Cohen, 2014a; Cohen & Donner, 2013; Nigbur et al., 2012), which refers to suppression of task-irrelevant information as means of interference resolution in general (Hanslmayr et al., 2008; Nigbur, Ivanova, & Stürmer, 2011). Second, with regard to attentional modulation of task-irrelevant information, we expected increased lateralization of sensory distractor processing (sensory lateralization effect) in theta frequency range for both response conflict (Pastötter & Frings, 2018) and stimulus conflict. Third, we presume that both stimulus and response conflict rely on selective attentional processing of task-relevant information. Thus, we expected to find a relative theta power decrease in conflict compared to non-conflict trials (i.e., a *negative theta effect*) over parietal sites, i.e., a target amplification effect in theta power.

## Methods

### Participants

Forty-one students from the University of Trier, Germany, were included in the study (36 women, one left-handed, mean age = 24.66 years, SD = 3.69 years). Three additional participants were tested but eliminated from analysis as they were RT-distribution far-outs with respect to Mosteller & Tukey (1977). All participants reported normal or corrected-to-normal vision and no participant reported any history of neurological disease. All participants gave written, informed consent before examination and received course credit for participation. The study was conducted in accordance with the Declaration of Helsinki and approved by the local ethics review committee at the University Trier.

### Procedure

**Fig 1.**
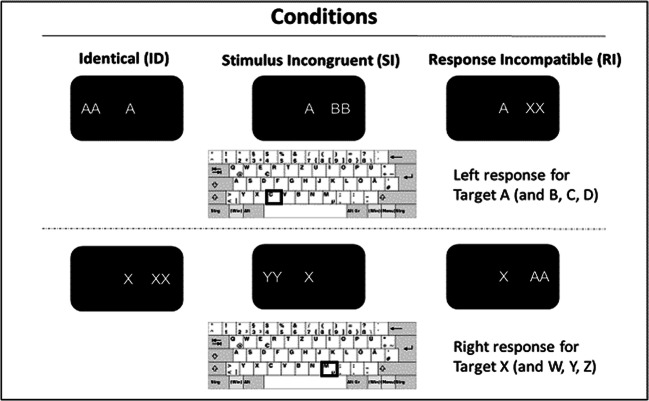
Experimental conditions of the lateralized Eriksen flanker task. Target letters were presented at central fixation (target A in the example above and target X below). Flanking distractor letters were presented either left or right to the targets. Participants were asked to respond with a left (target A, B, C, D) or right (target X, W, Y, Z) key press with their left or right index finger, respectively. In the ID condition, targets and distractors were identical. In the SI condition, targets and distractors were stimulus incongruent but response compatible. In the RI condition, targets and distractors were stimulus incongruent and response incompatible.

Participants performed a lateralized Eriksen flanker task, in which target letters were presented at central fixation and flanking distractor letters were presented either left or right to the target (Pastötter & Frings, 2018). In each trial, two distractor letters were shown. Target and distractor stimuli were the letters A, B, C, and D, which were mapped to a left-hand response, and the letters W, X, Y, and Z, which were mapped to a right-hand response. Three experimental conditions were realized: Target and distractors are either identical (ID), response compatible but stimulus incongruent (SI), or stimulus incongruent and response incompatible (RI). Stimulus (in)congruency (i.e., stimulus conflict) effects were examined by comparing data between congruent and incongruent trials in the ID and SI conditions, whereas response (in)compatibility (i.e., response conflict) effects were measured by comparing response compatible and incompatible trials in the SI and RI conditions (see Fig. 1). Note, that stimulus conflict and response conflict effects cannot be directly compared in this experimental design because both conflict effects lack an orthogonal realization by both relying on the SI condition.

All stimuli were shown in white on black background, at viewing distance of 65 cm. Stimuli were approximately 1.36 cm in size. Letters were written in Arial font. The target was presented in the centre of the screen, while the distractors were centred 3.5° to the left or right of the target. Between displays, a fixation cross was shown in the centre of the screen, which was 0.5° in size. Two keys (C and M) were marked on a standard QWERTZ keyboard, and participants were instructed to place their left index finger on C and their right index finger on M. The instructions asked them to press the C key in response to the target letters A, B, C, and D, and the M key in response to the target letters W, X, Y, and Z. Participants were asked to respond as quickly and accurately as possible to the target and to ignore the flankers; they should maintain their fixation on the centre of the screen and not move their eyes.

**Fig 2.**
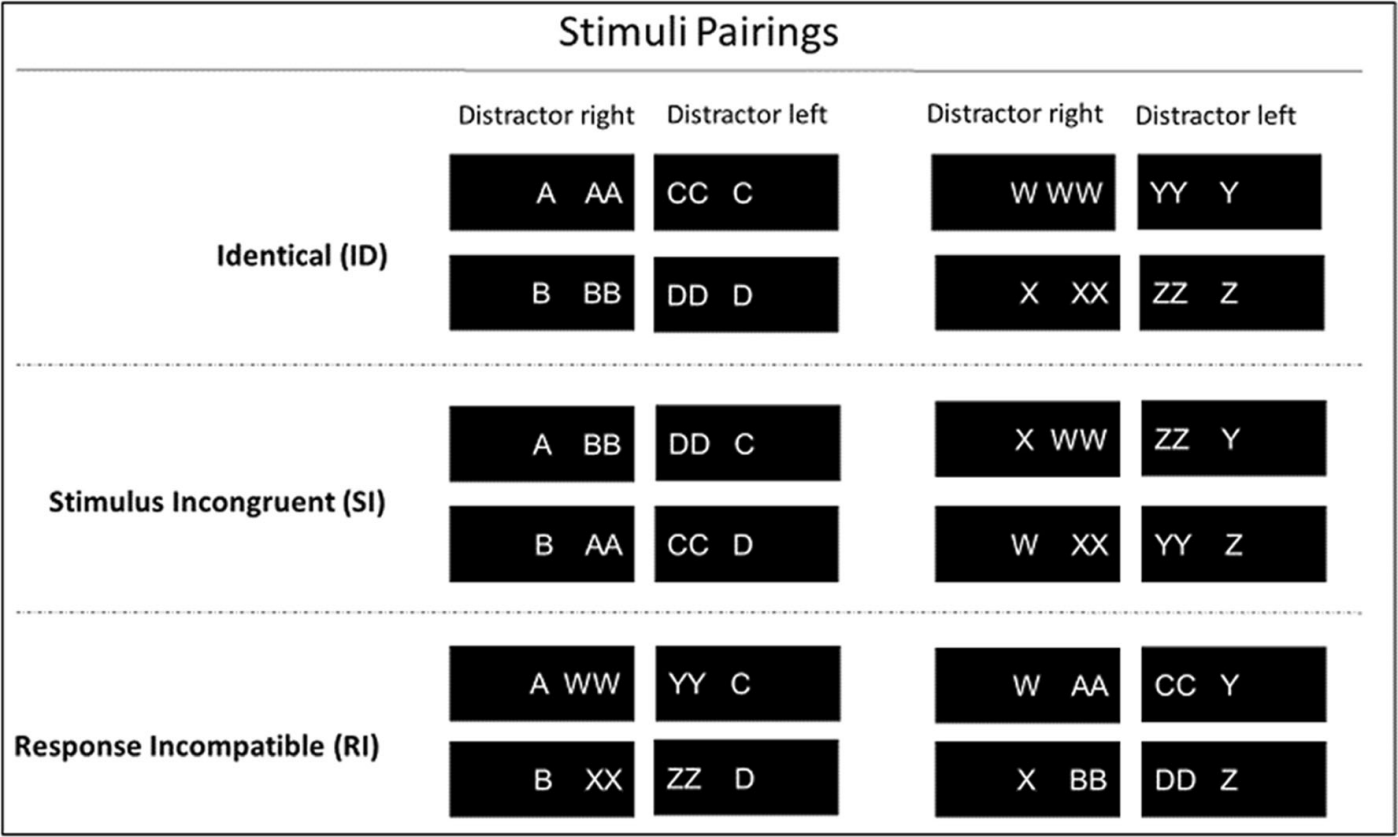
Predefined combinations of target-distractor pairings per condition. Letters presented in the centre of the screen are targets, whereas laterally presented stimuli are distractors. Each condition consisted of 288 trials, so that every target-distractor combination was repeated 36 times for each participant. Overall, every distractor stimulus was presented 18 times on the left and 18 times on the right hemifield-presentation side

An experimental session consisted of 864 single trials, 288 trials per condition, which means every target-flanker combination was presented 36 times during the experiment. The experiment consisted of six blocks, presenting 144 trials in each block, after each of which participants took a self-paced short break. All predefined combinations of target and distractor stimuli (Figure 2), with the same number of left- and right-hemifield distractor presentations, were realized in a counter-balanced manner. Order of ID, SI and RI trials was randomized across all participants with the constraints that no trial type, target letter, distractor hemifield presentation or reaction type were repeated more than three trials in a row.

Each single trial began with a fixation cross, which was shown in the centre of the screen for an interval of variable duration of 2–2.4 s. After presentation of the fixation cross, the target letter was shown together with two distractor letters. The target was shown in the middle of the screen, and the distractors were shown either left or right to the target. Target and distractor stimuli remained on the screen until key press. No feedback was provided in the experimental session. After the key press the next single trial started with presentation of the fixation cross. To familiarize participants with the procedure, they were given one block of 24 practice trials with feedback before the experiment. The experimental session of the Eriksen flanker task took about 36 minutes. Presentation and recording of behavioural responses were done with E-Prime software (v2.0, Psychology Software Tools).

### Analysis of behavioural data

Both mean RT and error rates were analysed in JASP (version 0.13.0.0). For RT analysis, only trials were included for which responses on both the current trial (*n*) and the previous trial (*n–1*) were correct. Single trials with reaction times greater than 1 s were excluded from the analysis. First, behavioural data were analysed with repeated-measures ANOVAs with the factor of experimental condition (ID, SI, RI) and the dependent variables RTs and accuracy rate. Greenhouse-Geisser correction was applied where appropriate. Next, paired samples *t*-tests were conducted to evaluate the significance of the stimulus conflict effect (difference between ID trials and SI trials) and the response conflict effect (difference between SI trials and RI trials).

### Recording of EEG data

Electrophysiological data were recorded from 65 Ag/AgCl electrodes, which were positioned according to the 10–10 electrode system with reference to FCz (EC80, Montage No. 1, Easycap). The ground was placed at location AFz. The electrooculogram (EOG) was recorded from four bipolar channels, positioned on the inferior and superior regions of the left eye and the outer canthi of both eyes to monitor the vertical and horizontal EOG. Electrode-skin impedance was kept below 5 kΩ for all electrodes. Signals were digitalized with a sampling rate of 500 Hz and amplified between 0.016 and 250 Hz (BrainAmp, BrainVision Recorder, v1.20, BrainProducts).

### Pre-processing of EEG data

EEG recordings were re-referenced offline against average reference and EOG corrected by using calibration data and generating individual EOG artifact coefficients, as implemented in BESA Research (v7.0, BESA Software). Remaining artifacts were marked by visual inspection. EEG data were segmented into epochs ranging from −2.5 to 2.5 s around the onsets of stimuli and responses. To avoid filter artifacts at the edges of the segments, further analyses were restricted to intervals ranging from −1.5 to 1.5 s around stimulus and response onsets, respectively. Segments containing artifacts and segments with response errors either on the current (*n*) or on the previous trial (*n-1*) were discarded from further analysis. For response-locked analysis, on average, 254 ID trials (SE = 2.22), 255 SI trials (SE = 2.55), and 252 RI trials (SE = 2.83) went into cluster analysis after artifact correction. For stimulus-locked analysis, on average, 255 ID trials (SE = 2.58), 256 SI trials (SE = 2.71), and 252 RI trials (SE = 2.72) went into cluster analysis. For SLI analysis, on average, 128 ID_left hemifield_ trials (SE = 1.29), 127 ID_right hemifield_ trials (SE = 1.39), 129 SI_left hemifield_ trials (SE = 1.44), 127 SI_right hemifield_ trials (SE = 1.36), and 126 RI_left hemifield_ trials (SE = 1.37), 126 RI_right hemifield_ trials (SE = 1.54) went into repeated measures ANOVA.

### Spectral EEG analysis

The EEG data were transformed into the time-frequency domain using a demodulation algorithm, which is implemented in BESA Research (v7.0). The algorithm consists of a multiplication of the time domain signal with a periodic exponential function, having a frequency equal to the frequency under analysis, and subsequent low-pass filtering. The low-pass filter is a finite impulse response filter of Gaussian shape in the time domain, which is related to the envelope of the moving window in wavelet analysis. The data were filtered in a frequency range from 2 to 30 Hz. Time resolution was set to 78.8 ms (full power width at half maximum; FWHM), and frequency resolution was set to 1.42 Hz (FWHM). Time-frequency data were exported in bins of 50 ms and 1 Hz. Both stimulus- and response-locked power changes were calculated, time-locked to stimulus or response onset, respectively. Stimulus- and response-locked changes in power were determined by calculating the temporal-spectral evolution, that is, power changes for all time-frequency points with power increases or decreases at time point *t* and frequency *f* related to mean power at frequency *f* over a preceding baseline interval (Pfurtscheller & Aranibar, 1977). Stimulus-locked power changes were determined in relation to a pre-stimulus baseline interval that was set from −250 to 0 ms time-locked to stimulus onset, whereas response-locked power changes were determined in relation to a baseline interval that was set from −1,250 to −1,000 ms time-locked to response onset (Pastötter & Frings, 2018).

### Analysis of response-locked effects

Time-frequency characteristics of response-locked effects in theta power (3-8 Hz) around response onset (−200 to 200 ms) were examined with permutation-based cluster analysis (BESA Statistics, v2.0, BESA Software), separately for stimulus conflict (ID vs. SI) and response conflict (RI vs. SI). For each contrast, a nonspatial cluster analysis was calculated first and a spatial analysis was calculated second. In the nonspatial cluster analysis, time-frequency spectrograms of response-locked power changes were averaged across the 65 electrodes and contrasted between conditions. Two-tailed *t*-tests were calculated for all time-frequency points (9 [50-ms time bins] × 6 [1-Hz frequency bins]). The sum of *t* values of adjacent time-frequency points that fell below a *p* value of .05 in the *t*-test was calculated as a test statistic. Random permutation analysis was calculated based on 5,000 randomization runs. In each randomization run, time-frequency data of the two conditions were interchanged randomly for each participant and *t*-tests were calculated for each time-frequency point. At the end of each run, *t* values of adjacent time-frequency points that fell below a *p* value of .05 were summed and the cluster with the highest sum of *t* values was kept. By these means, a null distribution of cluster sums was created from the 5,000 permutation runs, and the critical *p*_*crit*_ value for an empirically derived time-frequency cluster was estimated (Maris & Oostenveld, 2007; Sassenhagen & Draschkow, 2019). Next, empirical clusters with a *p*_*crit*_ value below .05 went into spatial analysis. For each cluster, power changes were averaged across data points of the cluster's maximum time range and maximum frequency range, separately for each electrode. These data were contrasted between conditions. Two-tailed *t*-tests were calculated for all electrodes. Spatial topographies were identified by considering those electrodes that fell below a *p* value of .05 in the *t*-test. No additional cluster analysis was calculated. Thus, both clustered and scattered effects of conditions were considered in the spatial analysis.

### Analysis of stimulus-locked effects

Time-frequency characteristics of stimulus-locked effects in theta power (3-8 Hz) after stimulus onset (0-500 ms) also were examined with permutation-based cluster analysis (BESA Statistics, v2.0, BESA Software), separately for stimulus conflict (ID vs. SI) and response conflict (RI vs. SI). For each contrast, a nonspatial cluster analysis was calculated first and a spatial analysis was calculated second. In a first step, paired *t*-tests for all time-frequency points (11 [50-ms time bins] × 6 [1-Hz frequency bins]) were calculated, averaged across topography, and clusters of contiguous data points that fell below a *p* value of .05 in the single *t*-tests were derived. For each empirical time-frequency cluster, the sum of *t* values of the single significant data points was kept as a test statistic. After 5,000 random permutations, the critical *p*_*crit*_ values for the empirically derived clusters were calculated. In a second step, spatial cluster analysis was used to examine the topography of the empirically significant clusters (*p*_*crit*_ ≤ .05) from the nonspatial cluster analysis. Spatial topographies were figured out by considering those electrodes that fell below a *p* value of .05 in the *t*-test.

### Analysis of sensory lateralization effects

Following Pastötter & Frings (2018), a sensory lateralization index (SLI) was calculated. First, for each of the 11 time bins from 0 to 500 ms, stimulus-locked theta power changes (3-6 Hz) were averaged across electrodes P5, P7, and PO7 for a left occipital region of interest (ROI) hemisphere, and across electrodes P6, P8, and PO8 for a right occipital ROI. Second, for each time bin, the SLI was calculated by subtracting mean theta power change of the ROI ipsilateral to distractor presentation side from mean theta power change of the ROI contralateral to distractor presentation side and averaging the difference value across left- and right-hemifield distractor presentations. For topographical illustration of the sensory-lateralization effect, SLIs were averaged from 50 to 250 ms following stimulus onset, independent of experimental condition. For statistical analysis of potential conflict effects, SLIs were calculated separately for each experimental condition and examined as a function of condition (ID, SI, RI) and time (11 time bins from 0 to 500 ms) via repeated measures ANOVA using JASP (version 0.13.0.0). Note that, a priori, lateralization effects also were analysed using cluster-based permutation analysis. In addition to the expected occipital clusters, several nonoccipital clusters, namely over midparietal and midcentral electrodes, were found to be significant in this analysis. Because we had no hypotheses regarding these nonoccipital clusters, we decided to not analyse theses data further and use the time-frequency setting and regions of interest from the Pastötter and Frings (2018) study for the analysis of sensory lateralization effects instead.

### Analysis of brain-behaviour correlations

To test for the relationship between behavioural data and electrophysiological data, Pearson’s rho correlations were computed for difference values of significant conflict effects (response conflict: RI minus SI; stimulus conflict: SI minus ID) between RT and stimulus-locked theta power (averaged across data points of a cluster’s maximum time range, maximum frequency range, and electrodes), response-locked theta power (averaged across data points of a cluster’s maximum time range, maximum frequency range, and electrodes), and theta SLI. Analyses were computed in JASP (version 0.13.0.0).

## Results

### Behavioural results

**Fig 3.**
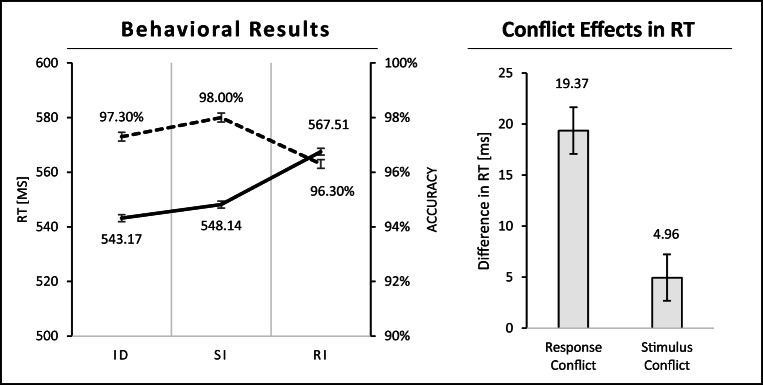
Behavioral results: RT and accuracy rates for each condition (ID, SI, RI); standard errors of the mean were corrected according to Cousineau (2005) for repeated-measures analyses. Conflict effects in RT: response conflict (RI minus SI) and stimulus conflict (SI minus ID) effects; standard errors of the mean

Mean RTs are depicted in Figure 3. A one-way repeated-measures ANOVA with the factor of experimental condition (ID, SI, RI) revealed a significant effect for RT, *F* (1.68, 67.27) = 64.05, *p* < .001, ω^2^ = .028. Significant conflict effects emerged in paired samples *t*-tests. Response conflict (RI minus SI) evoked on average 19.37 ms slower RT, *t* (40) = 9.19, *p* < .001, *d* = 1.435, and stimulus conflict (SI minus ID) evoked on average 4.96 ms slower RT, *t* (40) = 2.58, *p* = .013, *d* = 0.404.^1^

Accuracy rates are shown in Figure 3 as well. A one-way repeated-measures ANOVA with the factor of experimental condition (ID, SI, RI) revealed a significant effect for accuracy, *F* (1.48, 59.04) = 16.17, *p* < .001, ω^2^ = .102. A significant response conflict effect (1.66%) emerged in accuracy rates in a paired samples *t*-test, *t* (40) = 4.99, *p* < .001, *d* = 0.779; in contrast, no significant stimulus conflict in accuracy rates was found, *t* (40) < 1, one-sided.

### Physiological results

#### Results for response-locked analysis

**Fig 4.**
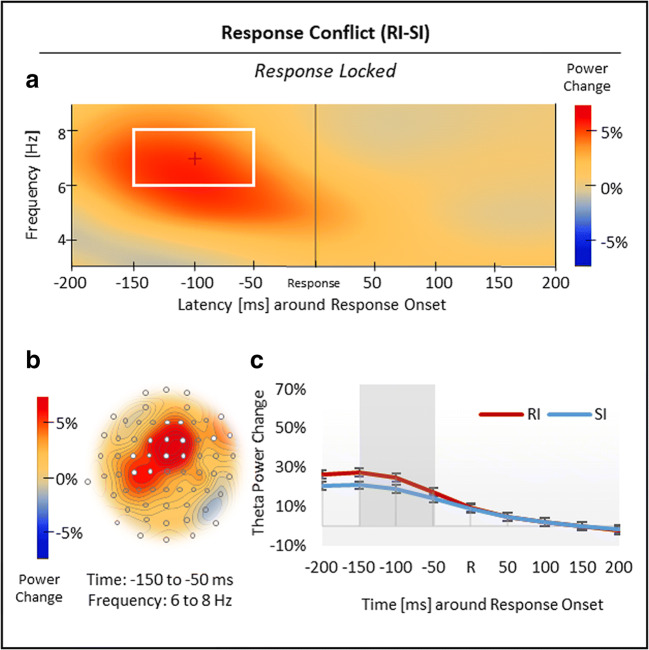
Results for the response-locked response conflict effect in theta power. (***A***) Regarding response conflict (RI vs. SI), nonspatial time-frequency analysis showed a positive theta effect, which was (***B***) most pronounced over midfrontal sites. (***C***) Time course of theta power change in RI and SI trials, time-locked to response onset R. Standard errors of the mean were corrected according to Cousineau (2005) for repeated-measures analyses. The white box (***A***), white electrodes (***B***), and the grey-shaded area (***C***) indicate significant effects

**Fig 5.**
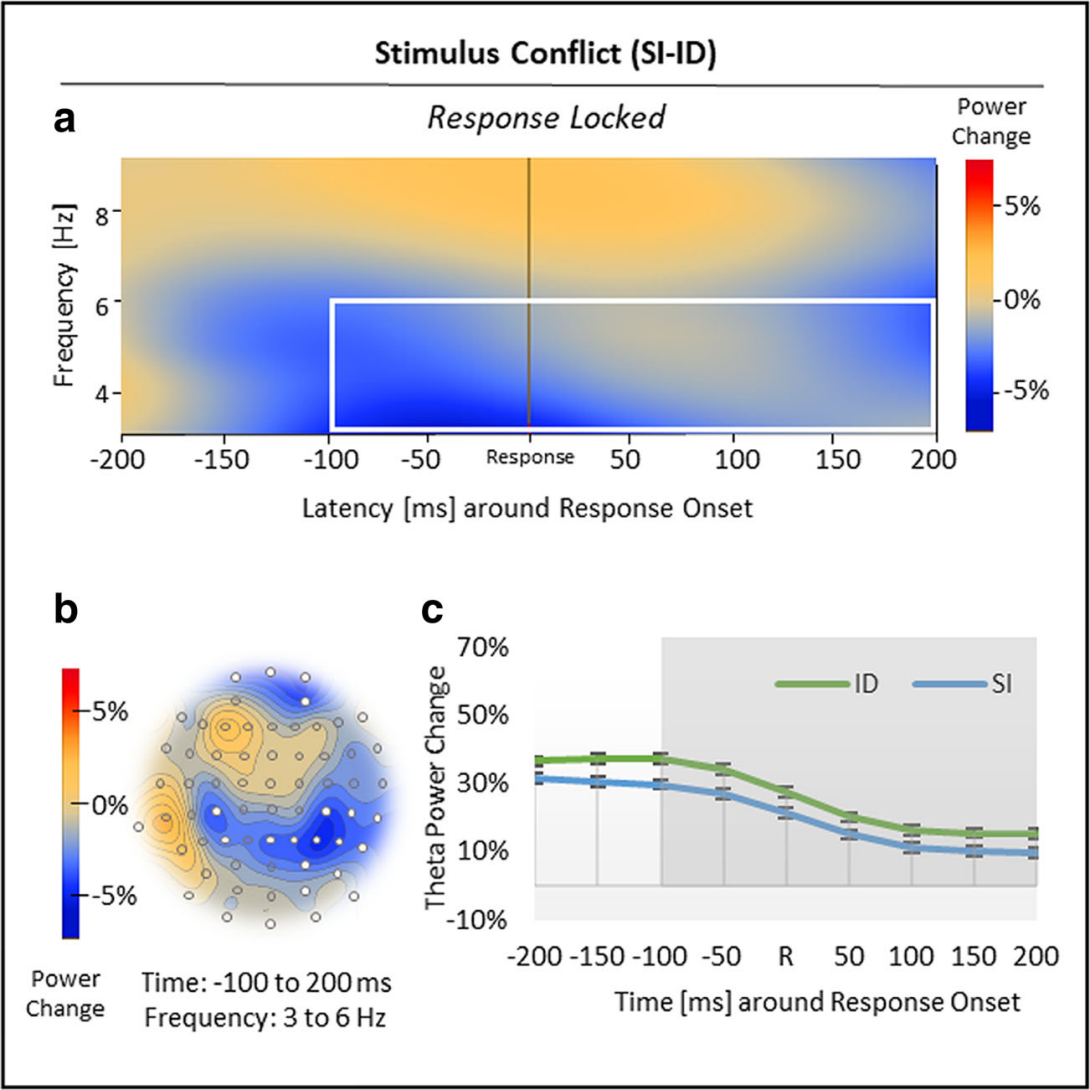
Results for the response-locked stimulus conflict effect in theta power. (***A***) Regarding stimulus conflict (SI vs ID), non-spatial time-frequency analysis showed a negative theta effect, which was (***B***) most pronounced over frontal and parietal sites. (***C***) Time course of theta power change in ID and SI trials, time-locked to response onset R. Standard errors of the mean were corrected according to Cousineau (2005) for repeated-measures analyses. The white box (***A***), white electrodes (***B***) and the grey-shaded area (***C***) indicate significant effects

Nonspatial cluster analysis, time-locked to response onset, revealed two significant clusters in total (Figures 4 and 5). First, with regard to response conflict (RI vs. SI), the analysis showed a larger increase in theta power (6-8 Hz) in RI compared with SI trials from −150 to −50 ms before response onset, *p*_crit_ < .001 (Figure 4A; see Figure 4C for time courses). Spatial analysis indicated that this *positive* theta effect was most pronounced over midfrontal sites (Figure 4B).

Second, with regard to stimulus conflict (SI vs. ID), nonspatial cluster analysis revealed a relative power decrease in theta power (3-6 Hz) in SI compared with ID trials from −100 to 200 ms around response onset, *p*_crit_ < .001 (Figure 5A; see Figure 5C for time courses). Spatial analysis indicated that this *negative* theta effect was located to frontal and parietal sites (Figure 5B).

#### Results for stimulus-locked analysis

**Fig 6.**
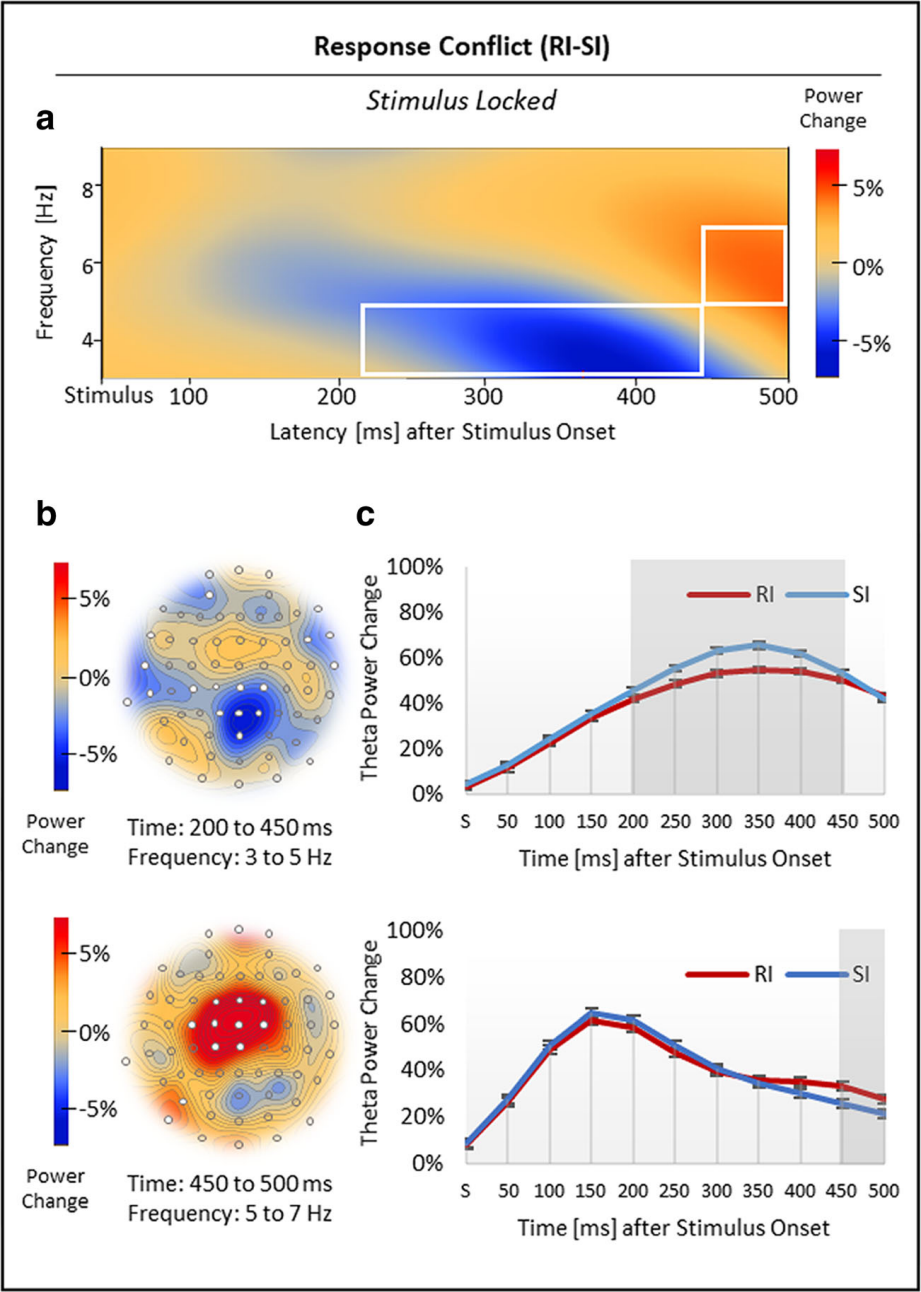
Results for the stimulus-locked response conflict effect in theta power. (***A***) Regarding response conflict (RI vs. SI), nonspatial time-frequency analysis showed a negative theta effect, which was (***B***) most pronounced over midparietal and temporal sites, followed by a positive theta effect in a midfrontal topography. (***C***) Time courses of theta power change in RI and SI trials, time-locked to stimulus onset S. Standard errors of the mean were corrected according to Cousineau (2005) for repeated-measures analyses. White boxes (***A***), white electrodes (***B***), and grey-shaded areas (***C***) indicate significant effects

**Fig 7.**
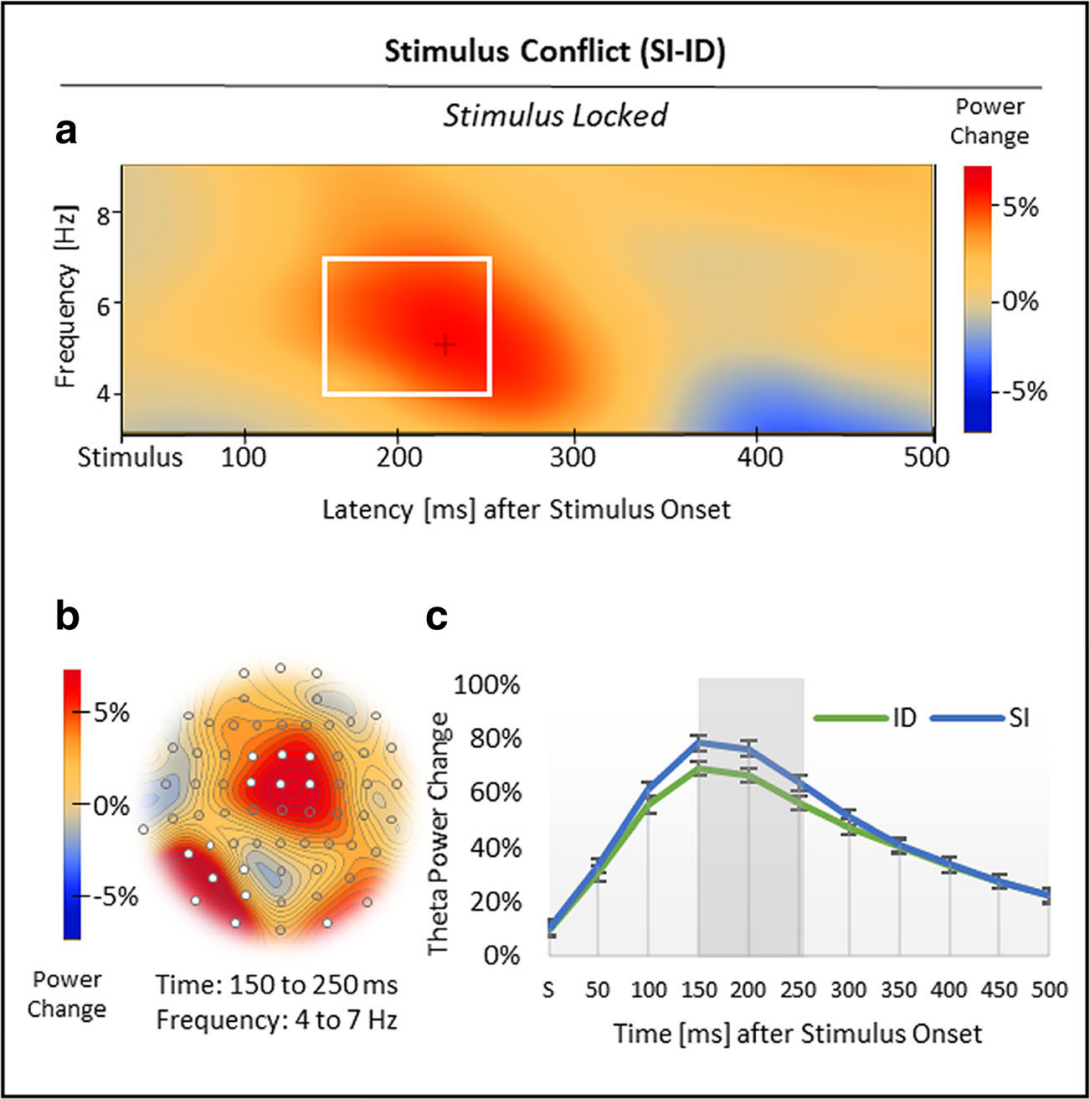
Results for the stimulus-locked stimulus conflict effect in theta power. (**A**) Regarding stimulus conflict (SI vs. ID), nonspatial analysis revealed a positive theta effect, which was (**D**) most pronounced over midfrontal and occipital sites. (**C**) Time course of theta power change in ID and SI trials, time-locked to stimulus onset S. Standard errors of the mean were corrected according to Cousineau (2005) for repeated-measures analyses. The white box (**A**), white electrodes (**B**) and the grey-shaded area (**C**) indicate significant effects

Nonspatial cluster analysis, time-locked to stimulus onset, showed three significant clusters in total (Figures 6 and 7). First, with regard to response conflict (RI vs. SI), the analysis revealed one cluster with relatively reduced theta power (3-5 Hz) in RI compared with SI trials from 200 to 450 ms after stimulus onset, *p*_crit_ < .001, and a second cluster with relatively increased theta power (5-7 Hz) in RI compared with SI trials from 450 to 500 ms after stimulus onset, *p*_crit_ < .001 (Figure 6A; see Figure 6C for time courses). Spatial analyses indicated that the first, *negative* theta effect was most pronounced over midparietal and frontotemporal sites, whereas the second, *positive* theta effect was clearly localized to midfrontal sites (Figure 6B).

Second, with regard to stimulus conflict (SI vs. ID), nonspatial cluster analysis revealed a cluster with relatively increased theta power (4-7 Hz) in SI compared with ID trials from 150 to 250 ms after stimulus onset, *p*_crit_ < .001 (Figure 7A; see Figure 7C for time courses). This *positive* theta effect was localized to midfrontal and (mainly left) occipital sites (Figure 7B).

#### Results for sensory lateralization

**Fig 8.**
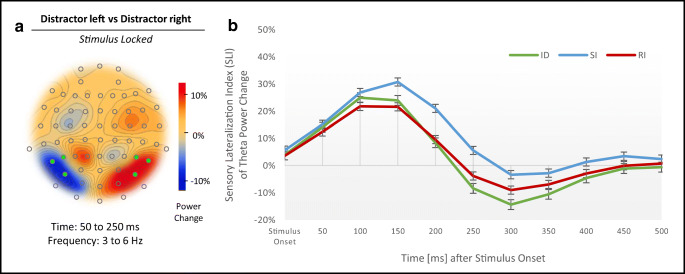
Results for sensory lateralization. (***A***) Topography of the sensory lateralization effect in occipital theta power (difference between left distractor presentation and right distractor presentation trials; 3–6 Hz; 50–250 ms). Green electrodes tag the predefined electrodes for further analysis. (***B***) Time course of the SLI across experimental conditions (ID, SI, RI) from 0 to 500 ms after stimulus onset. Standard errors of the mean were corrected according to Cousineau (2005) for repeated-measures analyses

Examination of predefined stimulus-locked theta power change (3-6 Hz) over left and right occipital sites from 50 to 250 ms after stimulus onset showed a clear sensory lateralization effect (Figure 8A). Repeated measures ANOVA (3 [conditions] x 11 [time bins from 0 to 500 ms]) of the SLI revealed a significant main effect of condition, *F* (2, 80) = 5.39, *p* = .006, ω^2^ = .018, a significant main effect of time, *F* (2.16, 86.46) = 15.88, *p* < .001, ω^2^ = .161, and a significant interaction between condition and time, *F* (4.92, 196.61) = 3.08, *p* = .011, ω^2^ = .008. In a second step, two repeated measures ANOVAs were conducted for response conflict (RI vs. SI) and stimulus conflict (SI vs. ID) independently, with the repeated factor time from 0 to 500 ms (11 [time bins]) and the difference in SLI between conditions as dependent variable. The main effect of time was significant for stimulus conflict, *F* (2.68, 107.14) = 4.41, *p* = .008, ω^2^ = .038, but not for response conflict, *F* (2.57, 102.86) = 2.50, *p* = .073, ω^2^ = .016. Follow-up one sample *t*-tests show that stimulus conflict modulated sensory lateralization significantly in a time window between 200 and 450 ms after stimulus onset, whereas response conflict influenced the SLI significantly from 150 to 250 ms after stimulus onset, but not in line with the postulated positive direction of the effect. Time courses of the SLI are shown in Figure 8B.

#### Results for brain-behaviour correlations

We correlated behavioural data (conflict effects in RT) with neural data (stimulus-locked and response-locked theta effects, SLI) to validate the result pattern (Table 1). No significant correlations emerged (all *p*s > .05) except from a negative correlation between the response conflict effect (RI vs. SI) in stimulus-locked theta power (*negative* theta effect averaged over eight midparietal significant electrodes) and the response conflict effect in RT, *r* = −.331, *p* = .034.^2^ The response-locked correlation between RT and the stimulus conflict effect (SI vs. ID) was only marginally significant, *r* = −.297, *p* = .059 (*positive* theta effect averaged over 16 parietal significant electrodes). Note that brain-behaviour correlations were not corrected for multiple comparisons.

**Table 1:** Brain-behaviour correlations

	Correlation coefficient *r*	Statistical significance *p*
Response-locked effects		
RT * positive theta effect (RI vs. SI)	−.242	.128
RT * negative theta effect (SI vs. ID)	−.297	.059
Stimulus-locked effects		
RT * positive theta effect (RI vs. SI)	.024	.882
RT * negative theta effect (RI vs. SI)	−.331	.034*
RT * positive theta effect (SI vs. ID)	−.150	.350
Sensory Lateralization Index		
RT * response conflict (RI vs. SI)	.018	.911
RT * stimulus conflict (SI vs. ID)	−.066	.683

Note: Positive theta effect resembles an increase in theta power, in contrast a negative theta effect resembles a decrease in theta power. For the negative theta effects, only the correlations between significant parietal sites and conflict effects in RT were listed. For the positive theta effects, only the correlations between significant midfrontal sites and conflict effects in RT were listed

Asterisk indicate a significant (*p* < .05) correlation

## Discussion

This study used a lateralized flanker task setting to examine brain oscillatory activities associated with conflict resolution in the theta frequency range (3-8 Hz). In general, the results support a domain-general conflict resolution account in a stimulus-driven conflict task, which relies on a combination of inhibition of distractors, depicted by *positive* theta power effects with a midfrontal topography and target amplification, depicted by *negative* theta power effects with a predominantly parietal topography.

### Summary of replicated results

Regarding behavioural measures, we observed stimulus incongruency effects in RT, as well as response incompatibility effects in RTs and accuracy rates, thus replicating earlier behavioural work (Notebaert & Verguts, 2006; Verbruggen, Notebaert, Liefooghe, & Vandierendonck, 2006). With regard to electrophysiological data, we replicated a robust *positive* response conflict effect (RI vs. SI) in response-locked and stimulus-locked theta power over midfrontal sites. Furthermore, a midfrontal and (mainly left) occipital stimulus conflict effect (SI vs. ID) emerged in stimulus-locked theta power, which unfolds in a time interval approximately 150 to 250 ms after stimulus onset. Together, these results are consistent with numerous findings of increased midfrontal theta power due to cognitive conflict (Cohen & Donner, 2013; Nigbur et al., 2012; Pastötter & Frings, 2018). Comparison of conflict effects leads to the suggestion that stimulus conflict processing precedes response conflict processing in time, reflecting the different demands of both conflict types: Perceptual incongruence in stimulus conflict is mainly processed in occipital and midfrontal areas, whereas response incompatibility is solely resolved in midfrontal cortices, as previous research confirms (for a review see Li et al., 2017).

### Target amplification in parietal theta

The cognitive conflict network consists of midfrontal, lateral frontal and parietal regions, which are functionally connected and assumed to communicate long-range conflict signals within the theta frequency range (van Driel et al., 2015). Our findings underpin and broaden the conflict network account by assuming selective attentional processing of task-relevant information to be represented in theta power as well. *Negative* theta effects emerged not only in stimulus-driven conflict (relative theta power decrease in SI vs. ID trials) in the response-locked analysis, but they also emerged in response-driven conflict (relative theta power decrease in RI vs. SI trials) in the stimulus-locked analysis. We suggest that increased parietal theta power in nonconflicting situations implies enhanced attentional processing of target features.

Because the EEG oscillatory analysis has a very good temporal resolution (Clayton, Yeung, & Cohen Kadosh, 2015), it is possible to observe the temporal sequence of conflict processing steps in the stimulus-locked results. First, early after stimulus onset, the cognitive system processes perceptual incongruency of distracting information (approximately 150-250 ms after stimulus onset) in occipital and midfrontal areas. Right after perceptual processing, the network focuses on attentional amplification of target information (approximately 200-450 ms after stimulus onset) in parietal and temporal cortices. Lastly, response execution inhibits response incompatible action plans, monitored by the ACC and reflected in midfrontal theta power. This timeline suggests that perceptual distractor suppression actually precedes parietal target amplification in time within intra-trial conflict processing. Note, however, that cluster-based permutation testing can diminish the temporal precision of processing onsets and offsets in favour of false-positive results (Sassenhagen & Draschkow, 2019). Therefore, the proposed time course of conflict resolution processing should be considered an approximation only.

Regarding the observed negative theta effect in the frequency range from 3 to 6 Hz, one can argue that the theta effect overlaps with the delta band (~2-4 Hz). In fact, in the cognitive control literature, there is evidence for conflict effects in the delta or “lower theta” frequency range (Cohen, 2014a; Riddle, Vogelsang, Hwang, Cellier, & D’Esposito, 2020; van Driel et al., 2015). Discussing control processing in the “lower theta” band, Ryman et al. (2018, p. 8) noted that “this lower frequency response is phenomenologically similar to classic frontal theta-band effects, suggesting that although these phenomena are technically delta band, they index a similar neural computation [as theta].” Besides, we note that interpretation of the exact frequency spectrum of reported effects must be handled with caution, since time-frequency compositions display blurred spectral boundaries due to permutation-based cluster analysis, sampling noise, and non-reproducible fluctuations in the signal-to-noise ratio (Cohen, 2014b).

It is notable that parietal target amplification (RI vs. SI) significantly explained variance in RT of response conflict, whereas midfrontal conflict processing did not reliably contribute to variance prediction in RT conflict effects. Positive, negative, and zero-order correlations between midfrontal theta and RT conflict effects have been reported in earlier work (Pastötter & Frings, 2018; Zavala et al., 2013). The present results suggest this ambiguity could be partially explained by considering the contribution of “negative” target amplification effects in the theta frequency range. Correlational analyses also revealed that only the parietal region of electrodes showing a target amplification effect correlated significantly with the response conflict effect in RT, whereas the frontotemporal electrodes showed no significant correlation. This result may lead to the conclusion that especially parietal cortex is involved in attentional modulation of behaviour, at least in the present flanker task. The target amplification effect in parietal theta power is in line with previous research that proposed parietal theta power indexing the initiation of attentional control in nonconflict tasks (Green & McDonald, 2008).

### Frontoparietal (theta) control network

The question arises how midfrontal cortex (ACC) and parietal cortex cooperate and manage conflict adaptively in a cognitive control network in theta power (Marek & Dosenbach, 2018). Several authors argued that theta phase synchronization signals the need for control adjustments in a conflict monitoring network, managed by the ACC in a hub-like manner (Botvinick et al., 2001; Cohen, 2014a; Liu et al., 2017). To fulfill this task, the ACC engages in interregional communication via theta synchronizations with task-related regions, such as the parietal cortex (Jiang, Bailey, & Xiao, 2018; Liu et al., 2017; Walsh, Buonocore, Carter, & Mangun, 2011). Vissers et al. (2018) reported interregional conflict-related communication between frontal and parietal sites in midfrontal theta power in the Simon task. Specifically, the authors observed that increased parieto-frontal phase synchronization reduced cognitive conflict, in dependence of sensory interference of task-relevant color features.

Based on the present results, we propose parietal cortex to specifically adjust attentional control to target information in situations of response-driven and stimulus-driven conflict. With regard to the early time course of target amplification processing, the present results challenge the notion of the conflict monitoring account (Botvinick et al., 2004) that ACC is the only structure to initiate the processing of cognitive control. The attentional amplification of target features, induced by parietal areas, might be a parallel process to interference management in the ACC, thus supporting the dual frontoparietal conflict network account (Marek & Dosenbach, 2018). In detail, the frontoparietal network supports control initiation, for instance biasing sensory information processing in visual cortices, and control adjustments in response to performance feedback. Marek & Dosenbach (2018) state that communication in the frontoparietal network via theta power (and lower alpha power; 4 Hz to 14 Hz) enables long-distance integration of conflict information, and especially top-down modulation of sensory networks, e.g. target and distractor information processing. Our data complement this idea by proposing that parietal cortex focuses attentional processing on target-related information, while distractor inhibition is managed by midfrontal brain structures in theta frequency. Going beyond popular conflict theories (Botvinick et al., 2004; Marek & Dosenbach, 2018), sensory lateralization analyses in our study suggest occipital cortex, not ACC, to initially detect cognitive conflict in perceptual incongruent situations, as Pastötter & Frings (2018) already showed. The authors reported a negative fronto-occipital correlation in theta power, which indicates that conflict processing in occipital structures even reduced conflict monitoring in the ACC.

In conclusion, the postulated frontoparietal (theta) control network might coordinate various brain structures to manage specific aspects of conflict, such as occipital areas for initial conflict detection (Pastötter & Frings, 2018), parietal cortex for target amplification, midfrontal ACC for conflict monitoring and the initiation of control adjustments (Cohen & Donner, 2013), and frontal cortex for further top-down allocation of attentional control (Lehr et al., 2019).

### Conflict driven modulation of sensory distractor processing

Theta oscillations in the visual cortex have been linked to lateralized processing in visuospatial attention, especially attentional distraction towards irrelevant sensory information (Pastötter & Frings, 2018; see also Appelbaum, Smith, Boehler, Chen, & Woldorff, 2011). Consistently, in the present study, we observed a sensory lateralization effect in theta power that referred to perceptual processing of lateralized distractor information in occipital and parietal brain areas. Laterally presented distractors received enhanced processing in the contralateral hemisphere, especially when the distractor had no perceptual feature overlap with the target stimuli in stimulus conflicting conditions. While finding a stimulus conflict effect in the lateralized processing of irrelevant distractor information, we did not replicate the modulation of sensory distractor processing by response conflict, as Pastötter and Frings (2018) suggested.

Experimental settings differed between the present study and the study by Pastötter and Frings (2018) in several aspects. For instance, eight different target letters (A, B, C, D, W, X, Y, Z) were used in the present study, whereas only four target letters (D, F, J, K) were used in the study by Pastötter and Frings (2018). This may have increased the variance of target presentations and thus increased attention to targets in the present study compared to the earlier work. In addition, Pastötter and Frings (2018) included two neutral distractor conditions (either neutral letters, which were not mapped to responses, or a neutral box were shown as distractors), whereas in all trials and conditions of the present study, distractors were letters and were mapped to responses. This may have reduced the variance of distractor type and the likelihood of conflict and thus reduced attention to distractors in the present study compared to the study by Pastötter and Frings (2018). Future studies are needed to investigate this in more detail. Importantly, the interesting question why we only observed a stimulus conflict effect on the sensory lateralization effect can be explained by theories relating to stimulus-response bindings and the role of attention for these bindings.

### TEC/BRAC

The idea that any kind of conflict resolution depends strongly on attentional and perceptual processing is linked to the theory of event coding (TEC; Hommel et al., 2001; Hommel, 2019) and the Binding-and-Retrieval-in-Action-Control framework (BRAC; Frings et al., 2020). There is already ample evidence that TEC provides a theoretical framework for midfrontal theta effects in conflict processing (Pastötter & Frings, 2018). In contrast to the notion of perceptual and response conflict-specific resolution techniques (Botvinick et al., 2001; Egner, Delano, & Hirsch, 2007), TEC and BRAC stress the perceptual and attentional adjustment mechanisms in conflict processing in general. According to action-related feature binding accounts (Frings et al., 2020), the selective attentional amplification of target features could solve not only perceptual conflict, but also response conflict as well, because all task-relevant or salient feature codes, such as the perceptual event, its required actions, and the task context, are automatically integrated in a common episodic event file. Our data support TEC and BRAC in the sense that parietal target amplification (*negative theta effect*) is found before response-driven conflict (RI vs. SI) and before the button press in stimulus-driven conflict (SI vs. ID). Therefore, one might assume that attentional processing (e.g., binding of stimulus features in an event file) might be the basis even for response incompatibility effects in a flanker task. This argumentation is complemented by approaches from Nigbur et al. (2015), which claim that enhanced sensory processing of target stimuli is essential for efficient stimulus and response conflict resolution.

The BRAC framework (Frings et al., 2020) might as well explain the conflict-driven modulation of the sensory lateralization effect based on attentional feature binding, e.g., grouping of distractive stimuli (Frings & Rothermund, 2011) or intentional/attentional weighting of (task-relevant) features (Hommel, Memelink, Zmigrod, & Colzato, 2014; Singh, Moeller, Koch, & Frings, 2018), which all lead to enhanced S-R binding. TEC (Hommel et al., 2001) also states that response conflict becomes reduced to its perceptual features after many repetitions of S-R episodes (Hommel, Proctor, & Vu, 2004), which might explain why we could not find a SLI modulation by response-driven conflict after averaging SLI results across 864 trials of S-R binding and retrieval in the current study. Future studies should investigate whether the processing of task-irrelevant information indeed shifts from response conflict to stimulus conflict over time.

## Conclusions

Previous research stressed the importance of attentional amplification of task-relevant stimuli in stimulus-driven conflict tasks (Nigbur et al., 2015) but did not evaluate further the neurophysiological underpinnings of target amplification effects. The present study examined the contribution of distractor inhibition and target amplification as means of conflict resolution techniques via theta oscillations. Our study shows that target-related (parietal) theta power can precede midfrontal theta power in (response) conflict situations. Therefore, the initiation of cognitive control might be driven by goal-directed processing of target information in parietal areas. The results support the notion of an interactive cognitive conflict network (Botvinick et al., 2001; Botvinick et al., 2004), including communicative exchange between ACC and parietal cortices, to manage stimulus and response conflict in an adaptive manner.

## Footnotes

1 No sequential modulation of conflict effects in trial *n* by conflict effects in trial *n-1* was observed, *F* (4,160) = 0.276, *p* = .893, neither for stimulus conflict, *F* (2,80) = 0.478, *p* = .622, nor for response conflict, *F* (2,80) = 0.079, *p* = .92. This finding is consistent with earlier findings by Verbruggen, Notebaert, Liefooghe, and Vandierendonck (2006), who also examined stimulus and response conflict effects in the Eriksen flanker task and found no sequential modulation.

2 The brain-behaviour correlation between response conflict in RT and the stimulus-locked negative theta effect (averaged across all significant electrodes including temporal clusters) failed to reach significance, *r* = −0.26, *p* = .101. For response-locked data, the correlation between stimulus conflict in RT and the negative theta effect (averaged across all clusters including frontal sites) was marginally significant, *r* = −0.269, *p* = .089.
